# Development of a Framework for Youth- and Family-Specific Engagement in Research: Proposal for a Scoping Review and Qualitative Descriptive Study

**DOI:** 10.2196/65733

**Published:** 2025-03-28

**Authors:** Sarah E P Munce, Clementine Jarrett, Vjura Senthilnathan, Dorothy Luong, Brooke Allemang, Katherine Bailey, Elaine Biddiss, Maria T Britto, Francine Buchanan, Christine Cassidy, Andrea Cross, Jessie Cunningham, Gina Dimitropoulos, Scott E Hadland, Monika Kastner, Tieghan Killackey, Kristina Kokorelias, Colin Macarthur, Samantha Micsinszki, Chavon Niles, F Virginia Wright, Alene Toulany

**Affiliations:** 1 Holland Bloorview Kids Rehabilitation Hospital Toronto, ON Canada; 2 Bloorview Research Institute Holland Bloorview Kids Rehabilitation Hospital Toronto, ON Canada; 3 Rehabilitation Sciences Institute University of Toronto Toronto, ON Canada; 4 Institute of Health Policy, Management and Evaluation University of Toronto Toronto, ON Canada; 5 The Canadian Coalition for Rights of Children Toronto, ON Canada; 6 Institute of Interdisciplinary Studies Carleton University Ottawa, ON Canada; 7 Feminist Institute of Social Transformation Carleton University Ottawa Canada; 8 Child Health Evaluative Sciences SickKids Research Institute Toronto, ON Canada; 9 KITE Toronto Rehabilitation Institute University Health Network Toronto, ON Canada; 10 The Hospital for Sick Children Toronto, ON Canada; 11 Factor-Inwentash Faculty of Social Work University of Toronto Toronto, ON Canada; 12 Temerty Faculty of Medicine University of Toronto Toronto, ON Canada; 13 Institute of Biomedical Engineering University of Toronto Toronto, ON Canada; 14 James M Anderson Center for Health Systems Excellence Cincinnati Children's Hospital Medical Center Cincinnati, OH United States; 15 Department of Pediatrics University of Cincinnati College of Medicine Cincinnati, OH United States; 16 SickKids Research Institute Toronto, ON Canada; 17 School of Nursing Dalhousie University Halifax, NS Canada; 18 Strengthening Transitions in Care Lab IWK Health Halifax, NS Canada; 19 CanChild Centre for Childhood Disability Research McMaster University Hamilton, ON Canada; 20 Department of Pediatrics McMaster University Hamilton, ON Canada; 21 School of Rehabilitation Science McMaster University Hamilton, ON Canada; 22 Health Sciences Library Hospital for Sick Children Toronto, ON Canada; 23 Faculty of Social Work University of Calgary Calgary, AB Canada; 24 Division of Adolescent and Young Adult Medicine Mass General for Children Boston, MA United States; 25 Department of Pediatrics Harvard Medical School Boston, MA United States; 26 North York General Hospital Toronto, ON Canada; 27 Toronto General Hospital Research Institute University Health Network Toronto, ON Canada; 28 Lawrence Bloomberg Faculty of Nursing University of Toronto Toronto, ON Canada; 29 Department of Occupational Science and Occupational Therapy University of Toronto Toronto, ON Canada; 30 Section of Geriatric Medicine Department of Medicine Sinai Health Toronto, ON Canada; 31 Department of Physical Therapy University of Toronto Toronto, ON Canada; 32 Department of Pediatrics University of Toronto Toronto, ON Canada; 33 ICES Toronto, ON Canada

**Keywords:** youth and family engagement, frameworks, implementation science, scoping review, qualitative

## Abstract

**Background:**

Youth and families play an indispensable role in health research, given their unique lived experiences and expertise. Aligning research with patients’ needs, values, and preferences can significantly enhance its relevance and impact; however, recent research has highlighted various challenges and risks associated with youth and family engagement in health research. These challenges encompass the perils of tokenism, power imbalances and dynamics, questioning the motives behind engagement, and limited accessibility to patient-friendly training for patient partners, as well as inadequate training on patient engagement for researchers and the absence of equitable engagement tools. To address these risks and challenges, different patient engagement models, theories, frameworks, and guiding principles have been developed and adopted; to date, however, their transferability to youth- and family-specific engagement in research has been limited.

**Objective:**

The objectives of this project are (1) to determine the extent of the literature on the application of patient engagement models, theories, frameworks, and guiding principles in the context of youth-specific research; and (2) to determine how meaningful the key components and constructs of these models, theories, frameworks, and guiding principles are to youth and their family members.

**Methods:**

This project will use an integrated knowledge translation approach and consists of 2 phases: (1) a scoping review to identify patient engagement models, theories, frameworks and guiding principles in youth research; and (2) a qualitative descriptive study using one-on-one semistructured interviews with youth and family members to understand their conceptualization of meaningful engagement in health research. For phase 1, the following databases were searched: Medline, CINAHL, EMBASE, PsycINFO, and the Cochrane Central Register of Controlled Trials. Literature from 2013 to August 28, 2024, was captured. Primary studies using a patient engagement in research model, theory, or framework, or guiding principles, in youth will be included. The risk of bias of included studies will not be assessed. Extracted data will be quantitatively summarized using numerical counts and qualitatively using content analysis. For phase 2, we will recruit 9 to 17 youth and 9 to 17 family members. Transcripts will be analyzed using an inductive approach outlined by Braun and Clarke.

**Results:**

The project has received funding from the Canadian Institutes of Health Research. A 9-member integrated knowledge translation panel consisting of 6 youth and 3 family members has been established.

**Conclusions:**

The findings from this study will identify what is currently known about the application of patient engagement models, theories, frameworks, and guiding principles in youth-specific research and the important components of these models, theories, frameworks, and guiding principles from the perspective of youth and their families. These findings will be instrumental to developing a youth- and family-specific engagement in research framework called the UNITE framework and subsequently, a validated measure.

**International Registered Report Identifier (IRRID):**

PRR1-10.2196/65733

## Introduction

### Background

Patients play an essential role in health research, given their unique lived experiences and expertise, which can significantly enhance the quality, relevance, and impact of research by aligning it with patient needs, values, and preferences [1,2]. The overarching objective of patient engagement in health research is to generate research that contributes to improved health care service delivery, clinical outcomes, and population health [3]. Recent research has highlighted the myriad benefits and positive outcomes associated with patient engagement for patient partners, investigators, and research endeavors [4-8]. For example, engaging patients has influenced initial research priorities, study designs, interpretation of findings, health care interventions, and knowledge mobilization strategies, resulting in outcomes that more closely align with patient perspectives [7,9]. When patient engagement is characterized as meaningful and authentic, it provides a rewarding experience for patients and researchers alike [10].

However, other recent research has highlighted various challenges and risks associated with patient engagement in health research. These challenges, which include tokenism [11-14], power imbalances/dynamics [12,15], limited accessibility to patient-friendly training for patient partners [16], and the absence of equitable engagement tools [17], can have lasting effects on patient partners, including mental and physical exhaustion, deteriorating health, doubts about the value of engagement, and a sense of having personally failed both the team and the broader patient community [18]. A recent article by patient partners led by Richards et al [18] on how patient engagement can falter highlighted key themes, including “patient partners as a checkmark,” “unconscious bias towards patient partners,” “lack of support to fully include patient partners,” and “lack of recognizing the vulnerability of patient partners” [18].

To address these challenges and risks, various patient engagement models, theories, frameworks, and guiding principles (eg, the strategy for patient-oriented research [SPOR], the patient engagement in research [PEIR] framework, and “ways community members can participate in the stages of research” from the Ontario Brain Institute) have been developed and adopted within the research community [10,19,20]. For example, the PEIR framework [10] includes eight key components, which collectively contribute to meaningful patient engagement in research: (1) procedural requirements, (2) convenience, (3) contributions, (4) support, (5) team interaction, (6) research environment, (7) feeling valued, and (8) benefits. A subsequent measure of meaningful patient engagement in research from the patient perspective has been developed, the Patient Engagement in Research Scale (PEIRS-22) [21,22]. This measure is designed to be completed by adult patients and family caregivers who partner with researchers on projects.

It is important to acknowledge that existing frameworks often predominantly emphasize the benefits of patient partners to the research project and team, sometimes overlooking the reciprocal benefits that may occur between the research team and patient partners, especially youth partners [23]. This skewed approach fails to fully consider the potentially extractive nature of research collaboration, as aptly described by Metz and Damschroder [23], where the research process can unintentionally exploit the knowledge and contributions of youth without adequately reciprocating in terms of personal and professional development opportunities. This issue takes on particular significance when crafting an engagement framework tailored for youth, as research teams have the potential to foster skill development, positively impact their life and career trajectories, and contribute to their holistic growth [23].

Despite the significant contribution of the PEIR framework [10] and the PEIRS-22 [21,22], they lack the incorporation of a comprehensive review of the evidence on existing frameworks, and their development was based on participants with limited diversity in terms of gender, race, education, primary diagnosis (all had arthritis), and age [10,21,22]. These limitations impede the transferability of this framework and measure to youth- and family-specific engagement in health research.

Furthermore, there is a model of engagement specifically designed for use with youth called the McCain model of youth engagement [24]. However, it was developed solely in the context of youth mental health systems research for youth and young adults aged 15 to 29 years [24], limiting transferability (ie, across different contexts and family members) and highlighting the need to develop a broader framework. Accordingly, our research seeks to directly address these specific gaps in existing engagement models, theories, and frameworks by developing a youth and family-specific engagement in research framework, the UNITE framework.

### Research Objectives

This proposal has two objectives: (1) to determine the extent of literature on the application of patient engagement models, theories, frameworks, and guiding principles in the context of youth- and family-specific research; and (2) to understand how meaningful the key components and constructs of these models, theories, frameworks, and guiding principles are to youth and their family members. Collectively, these findings will be foundational to the development of the UNITE framework and a subsequent validated measure.

## Methods

### Study Design

This study will be conducted in 2 phases, with phase 1 consisting of a scoping review and phase 2 consisting of a qualitative descriptive study.

### Integrated Knowledge Translation Approach

Integrated knowledge translation (iKT) is defined as a collaborative relationship between researchers and relevant knowledge users as partners that facilitates mutually beneficial decision-making related to a study or research program [25]. Youth aged 10 to 24 years in Canada and their family members who have engaged in health research (ie, as patient partners) in the last 3 years were recruited as iKT panel members via Holland Bloorview Kids Rehabilitation Hospital’s Youth Advisory Council and Family Leader Program, professional networks, social media pages, and email lists. A sample of diverse youth advisors who have participated in our previous research on best practices in youth engagement were also approached, including youth with disabilities or developmental differences [26]. A total of 6 youths and 3 family members were recruited to be part of the iKT panels. The lead youth representative will lead the iKT panels. The lead youth representative and research team decided to hold separate iKT panels (ie, having a youth panel and a family panel with the opportunity to mix panels when needed). This approach will ensure that both groups feel comfortable and can freely express their unique perspectives. Activities of the panels have included planning study activities, and may include participant recruitment, data collection and analyses, and knowledge mobilization. Panel discussions will be conducted in a manner that respects diverse perspectives and experiences. A reflective exercise on equity, diversity and inclusion developed by the Strategy for Patient-Oriented Research Evidence Alliance will be conducted with iKT panel members to encourage dialogue and understanding around equity, diversity and inclusion [27].

### Phase 1: Scoping Review on Patient Engagement Models, Theories, Frameworks, and Guidance in Youth Health Research

The methodology for the scoping review will follow the methodological frameworks of the Joanna Briggs Institute (JBI) [28] and Khalil et al [29]. The scoping review protocol has been registered on the Open Science Framework Registries [30] and was guided by and reported according to the Preferred Reporting Items for Systematic Review and Meta-Analysis Protocols (PRISMA-P) [31]. The completed PRISMA-P checklist can be found in Multimedia Appendix 1. The results of the scoping review will be reported using the Preferred Reporting Items for Systematic Reviews and Meta-Analysis extension for Scoping Reviews (PRISMA-ScR) checklist [32].

#### Stage 1: Developing a Search Strategy

The population, concept, and context (PCC) framework was used to guide the search strategy (population: World Health Organization [WHO] definition of “young people” as those aged 10-24 years [33]; concept: patient engagement in research; context: models, theories, frameworks, and guiding principles). With the assistance of a research librarian, literature search strategies using Medical Subject Headings and text words related to patient engagement and models, theories, frameworks, and guidance or guiding principles were developed. The search strategy combined structured database-specific subject headings (as available) and keywords or synonyms. An information specialist with expertise in conducting searches for systematic and scoping reviews drafted the search strategy using OVID Medline (Multimedia Appendix 2) and worked with the research team to refine and finalize the search. The final search strategy also underwent peer review using the Peer Review of Electronic Search Strategies (PRESS) statement checklist with another librarian/information specialist [34].

The following databases were searched: Medline, CINAHL, EMBASE, PsycINFO and Cochrane Central Register of Controlled Trials. Searches were limited to English. Literature from 2013 to August 28, 2024 was captured, consistent with when the term “patient engagement” became frequently used [35]. We will also search the gray literature in specialized databases like OpenGrey, Grey Literature Report, and GreyNet International, platforms like arXiv, bioRxiv, and SSRN, and databases such as ProQuest Dissertations and Theses.

#### Stage 2: Evidence Screening and Selection

All primary studies using a model, theory, or framework for patient engagement in research among young people will be eligible for inclusion. Studies with a model, theory, or framework may have also reported on guiding principles. We define young people as those aged 10 to 24 years, consistent with the WHO definition [33]. We define a model as the essential elements or variables of a phenomenon or a specific aspect of a phenomenon; a theory as “a set of analytical principles or statements designed to structure our observation, understanding and explanation of the world”; and a framework as an explanation of a phenomenon by organizing it into a collection of descriptive categories and the relationships between them [36]. Systematic reviews, meta-analyses, editorials, commentaries, and nonspecific conference proceedings will be excluded to focus on including primary results and not preliminary findings or ongoing research; however, the reference lists of such articles will be hand-searched for relevant articles.

Removal of duplicates as well as level 1 and level 2 screening will be managed through Covidence. To increase reliability, the level 1 screening form will be piloted on a random sample of approximately 50 articles. Eligibility criteria descriptions will be revised if deemed necessary by the team, or if low agreement (ie, <70%) [37] is observed, to improve the consistent application of the selection criteria. Agreement will be measured using Cohen κ [38]. A pilot test of the level 2 screening will also be performed on approximately 25% of the articles, similar to the process for level 1 screening. For studies that are excluded at level 2, the reason for exclusion will be recorded. All screening will occur in duplicate and independently. When necessary, another reviewer will be sought to resolve conflicts.

#### Stage 3: Data Extraction

A standardized data extraction form will be developed by the research team and iKT panels based on the *JBI Manual* data extraction recommendations [28] and those recommended for the extraction, analysis, and presentation of results in scoping reviews [28,39]. Extracted data will include study characteristics (eg, study design, year of publication, geographic location), youth and family participant characteristics (eg, age of the youth), and details of the engagement models, theories, frameworks, and guiding principles (eg, components or values and principles and how they were enabled or enacted in the study), as well as study results. Additional categories for data extraction identified through discussions with the research team and iKT panels will be added to the final data extraction template as applicable. The data extraction template will be piloted for 2 to 3 articles to ensure all relevant results are extracted. All data will then be extracted in duplicate by 2 independent reviewers. Discrepancies in the extracted data will be discussed and resolved by the 2 reviewers. Quality and risk of bias will not be assessed, as this is not required in scoping reviews [32].

#### Stage 4: Data Analysis

The extracted data will be quantitatively summarized using numerical counts and qualitatively summarized using content analysis [40]. The data will be grouped by the main components of the model, theories, frameworks, guiding principles (and how they were enabled or enacted), study designs, and associated methods (eg, one-on-one interviews). We will also synthesize data on how youth and family members were engaged throughout the research process, the types of outcomes collected, and results. Depending on the available data, subgroup analyses may be conducted by health condition, sex, gender-related variables, and other PROGRESS-Plus characteristics (PROGRESS-Plus is a term meaning “place of residence, race/ethnicity/culture/language, occupation, gender/sex, religion, education, socioeconomic status, social capital”) [41]. If feasible, we will contact the study authors of the included studies to confirm that all the data collected were included in the published article (ie, nothing was excluded due to word count limitations of a journal).

### Phase 2: Qualitative Study to Understand Key Components and Constructs for Development of a Framework for Youth- and Family-Specific Engagement in Research

#### Study Design

Phase 2 will adopt a qualitative descriptive approach [42,43]. Findings from the scoping review will inform the development of the interview guide for phase 2, such as identifying what aspects of the included models, theories, frameworks, and guiding principles resonate with youth and family members and which do not.

#### Recruitment

We will recruit Canadian youth (aged 10-24 years) and their family members who have had experience in youth engagement in health research (eg, as partners of a research project) within the last 3 years. Participants will be recruited via our professional networks, including those of the iKT panel members (eg, the Kids Brain Health Network, the Bloorview Research Institute Family Engagement Office, and the SickKids Patient and Family Engagement Office), social media pages, and email lists. We will also enlist the help of a diverse group of youth advisors who have participated in our research team’s previous research for recruitment [26,44,45]. We aim to recruit 9 to 17 youth and 9 to 17 family members; this is consistent with the sample size in qualitative studies to reach thematic saturation [46].

#### Data Collection

Youth and family participants will take part in separate, one-on-one, semistructured telephone or online interviews with a member of the research team with expertise in qualitative research methods [47,48]. All interviews will be digitally recorded and transcribed verbatim for data analysis. The interview guides will consist of questions focused on youth and family members’ conceptualization of meaningful engagement in health research. The interview guide will be pilot-tested with various members of the research team and iKT panel. We will use probes or recursive questioning during interviews to explore issues in greater depth and verify the interviewer’s understanding of the collected information [49,50].

#### Data Analysis

We will use inductive thematic analysis, as described by Braun and Clarke [51,52], which is consistent with the pragmatic orientation of this research [53]. Codes and themes will be refined through discussion with the larger research team. The software program NVivo (version 14; Lumivero) will be used during the analysis of the transcripts to help organize the codes. Multiple aspects of trustworthiness will be used [54]. For example, we will demonstrate credibility via peer debriefing with various members of the research team and the iKT panels. Transferability will be accomplished by describing the study samples. Independent analysts will review the data and contest the themes to ensure dependability. Finally, confirmability will be accomplished by providing decision trails between data and interpretation [54].

#### Ethical Considerations

Ethics approval for phase 2 of this project will be obtained from the first author’s primary institution (Holland Bloorview Kids Rehabilitation Hospital, Bloorview Research Institute). Informed consent will be obtained from participants prior to the start of the interview. Transcripts from the interviews will be deidentified to ensure privacy prior to data analysis. Participants will be compensated for their time in the form of gift cards. The amount compensated will be consistent with the Canadian Institutes of Health Research (CIHR) Patient Partner Compensation Guidelines (CAD $50 [US $34.96] per participant) [55].

## Results

This work is supported by a CIHR Healthy Youth Catalyst Grant received in March 2024 (HEY- 192883; Multimedia Appendix 3). A 9-member iKT panel consisting of 6 youth and 3 family members has been established and has been actively involved in the study. We anticipate that phase 1 of the study will be completed in March 2025. Ethics approval for phase 1 was not required as it did not involve collecting or using data from participants. Ethics approval for phase 2 of the study will be applied for in winter 2025. Phase 2 of the project is anticipated to be completed in August 2025. A detailed timeline of the project can be found in Multimedia Appendix 4.

## Discussion

### Anticipated Findings

The findings from this study will allow us to identify what is currently known about the application of patient engagement models, theories, frameworks, and guiding principles, which are often designed for adults, in the context of youth-specific research, and to understand the importance of the components that make up these models, theories, and frameworks from the perspective of youth and their families.

### Dissemination Plans

We will use a variety of passive and active end-of-grant knowledge mobilization approaches to disseminate our findings, which will be codeveloped with our iKT panels. We will ensure youth and family voices are heard to develop flexible communication plans that will suit diverse needs. Traditional knowledge translation will include dissemination through meetings locally, nationally, and internationally (eg, PxP and For Patients, By Patients) and publications in peer-reviewed journals. Members of the research team are affiliated with and situated within pediatric institutions where the UNITE framework will be disseminated and implemented. We will codevelop plain language summaries with youth and family partners with clear, simple, and individualized messages for patients and family and community service organizations to augment the accessibility of the information. Finally, members of the research team will also discuss and distribute the UNITE framework within their expansive training curriculum and a planned youth-focused engagement in research course.

### Future Directions

This project lays the foundational work for developing a patient engagement framework called the UNITE framework, which will include equity, diversity, and inclusion considerations, and the subsequent development and validation of an associated measure of engagement. Future research will involve the implementation of the UNITE framework and a subsequent measure in a learning health system context within pediatric institutions that our research teams are associated with. The UNITE framework and associated measure will contribute to meaningful and sustained engagement of youth and their families in health research by addressing gaps in current patient engagement frameworks.

### Strengths and Limitations

A strength of this study is the inclusion of gray literature (eg, reports and policy literature) for phase 1 (the scoping review). This will provide a more comprehensive understanding of the concept (patient engagement in research) and context (models, theories, frameworks, and guiding principles) that we are interested in, as well as mitigate publication bias [56]. In addition, the search strategy for phase 1 has been reviewed by the iKT panel and undergone peer review using the PRESS statement checklist [34], which further strengthens the relevance, comprehensiveness, and quality of the search strategy. One limitation of this project is the potential for selection bias (specifically for phase 2), where individuals who had either very positive or very negative experiences with patient engagement in research may be more likely to participate in the study, which may limit the applicability of the study’s findings. However, the adoption of the iKT approach and having the iKT panels (consisting of youth and their families) assist in the recruitment for phase 2 of the study should result in the recruitment of a variety of individuals with diverse interests.

### Conclusions

The current proposal will lead to the development of a youth- and family-specific engagement in research framework, UNITE, with future research focused on the development of an associated validated measure. The UNITE framework and measure will lay the foundation for meaningful and sustained engagement in health research by youth and their families, ultimately contributing to enhanced health care service delivery, improved clinical outcomes, and increased overall well-being and quality of life for youth and their families [1-3].

## Appendix

Multimedia Appendix 1 PRISMA-P checklist.

Multimedia Appendix 2 Medline search strategy.

Multimedia Appendix 3 Peer-review report by the Catalyst Grant: Healthy Youth competition, Canadian Institutes of Health Research (Canada).

Multimedia Appendix 4 Timelines and milestones for the youth- and family-specific engagement in research (UNITE) project.

## Abbreviations

CIHR: Canadian Institutes of Health Research
iKT: integrated knowledge translation
PCC: population, concept, and context
PEIR: patient engagement in research
PEIRS-22: Patient Engagement in Research Scale-22
PRESS: Peer Review of Electronic Search Strategies
PRISMA-P: Preferred Reporting Items for Systematic Review and Meta-Analysis Protocols
PRISMA- ScR: Preferred Reporting Items for Systematic Reviews and Meta-Analysis Extension for Scoping Reviews
SPOR: strategy for patient-oriented research
WHO: World Health Organization

## Data Availability

The data collected during this study are available from the corresponding author upon reasonable request.
