# Supplementary material for: Development of a Framework for Youth- and Family-Specific Engagement in Research: Proposal for a Scoping Review and Qualitative Descriptive Study
Source: JMIR Res Protoc. 2025 Mar 28;14:e65733. doi: 10.2196/65733 (PMC11992488; doi:10.2196/65733)
Supplement: Multimedia Appendix 2 [file resprot_v14i1e65733_app2.pdf]

Appendix 2 –Medline Search Strategy

Ovid MEDLINE(R) Epub Ahead of Print and In-Process, In-Data-Review & Other Non-Indexed Citations and Daily

#	Searches	Results
1	*Patient Participation/ or ((patient? or child* or youth* or adolescen* or teen* or "patient and family" or "PFE" or "patient/caregiver" or caregiver? or carer? or guardian* or family* or families or parent* or mother* or father* or stakeholder? or client* or "care partner*") adj2 (particip* or partner* or engag* or perspective* or advisor* or activat* or empower* or involv* or collab* or consult*)).tw,kf.	308954
2	Professional-Family Relations/ or *"Patient-Centered Care"/ or "Translational Science, Biomedical"/ or (("Patient-Centered*" or "Patient-oriented" or "patient-centred*" or "patient-centric" or "child-centered" or "child-centr*" or "youth-centered*" or "youth-oriented" or "youth-centr*" or "person-centred" or "person centric" or "person oriented" or "family-centric" or "family-oriented" or "family centred*" or "family centered*" or "user centric" or "user oriented" or "client oriented" or "client centric" or "client oriented" or "client centred" or "client centered" or "person-centered" or "user centered" or "user centred" or "knowledge user*" or "co-design*" or "co-creat*" or "co-produc*" or partner* or "patient partner*") adj2 (research* or iKT or "integrated knowledge translation" or "integrated knowledge transfer*" or "disseminat*" or "knowledge mobili?ation*" or KM)).tw,kf.	36642
3	"Advisory Committees"/ or ("advisory committee" or "family advisory committee" or "patient and family advisory committee" or "youth participatory action research" or YPAR).tw,kf.	16441
4	("patient and public involv*" or "Public and Patient Engagement").tw,kf.	2019
5	3 or 4	18443
6	exp adolescent/ or young adult/ or (adolescen* or youth* or young adult? or young people or young person? or juvenile? or minor or minors or teen* or teen-age* or highschool* or high-school*).tw,kf.	3264798
7	5 and 6	2076
8	1 or 2 or 7	340692
9	"Community-Based Participatory Research"/ or ((CEPB or "Community-Based Participatory" or "Consumer Driven Community Based" or "participatory action") adj2 research*).tw,kf.	10218
10	8 and 9	2370
11	((partnership* or research* or study or studies or pragmat*) adj2 (psychometr* or "guiding principle*" or engag* or "guiding value*")).tw,kf.	10591
12	8 and 11	2161
13	((partnership* or research*) adj2 (Engag* or measur* or framework* or theor* or model* or "ground rule?" or "Terms of Reference")).tw,kf.	37555
14	8 and 13	3166
15	"Diversity, Equity, Inclusion"/ or (Diversi* or Equit* or Inclusi* or EDI* or DEI*).tw,kf.	1184404

Appendix 2 –Medline Search Strategy

16	8 and 15	23757
17	10 or 12 or 14 or 16	28423
18	*Health status/ or ((health* or wellbeing or well-being) adj2 (Level* or overall* or status* or quality or knowledge or attitude? or practice?)).tw,kf.	313306
19	17 and 18	1970
20	mental health services/ or chronic disease/ or (mental health* or mental* ill*).tw,kf. or (chronic* adj2 (disease* or ill* or condition? or disorder?)).tw,kf.	892675
21	17 and 20	3464
22	"Health Behavior"/ or ("health* behavio?r*" or "smoking cessation*" or "food related intervention*" or (pregnan* adj2 intervention)).tw,kf.	114994
23	17 and 22	539
24	19 or 21 or 23	5403
25	limit 24 to (english language and yr="2013 -Current")	4452
26	remove duplicates from 25	4430
