# Supplementary material for: Development of a Framework for Youth- and Family-Specific Engagement in Research: Proposal for a Scoping Review and Qualitative Descriptive Study
Source: JMIR Res Protoc. 2025 Mar 28;14:e65733. doi: 10.2196/65733 (PMC11992488; doi:10.2196/65733)
Supplement: Multimedia Appendix 3 [file resprot_v14i1e65733_app3.pdf]

Review Type/Type d'évaluation: Committee Member 1/Membre de comité 1
Name of Applicant/Nom du chercheur: Munce, Sarah Elizabeth
Application No./Numéro de demande: 513340
Agency/Agence: CIHR/IRSC
Competition/Concours: 2023-11-08 Catalyst Grant: Healthy Youth/Subvention catalyseur : Jeunes en santé
Committee/Comité: Catalyst Grant : Healthy Youth/Subvention catalyseur : Jeunes en santé
Title/Titre: Youth and Family Specific Engagement in Research (UNITE): A Framework Development Project

---

Assessment/Évaluation:

A. An assessment of the proposal based on the evaluation criteria in the funding opportunity

This project highlights the importance of including patient perspectives in the healthcare system. The project clearly states its objectives and outlines the first phase, which is a scoping review, followed by qualitative interviews. Although this proposal follows common approaches to involving patients, it would have been beneficial to see innovative methods for engaging with youth.

While a mixed methods approach is employed, there is limited discussion about the theoretical foundation of the proposal. The scoping review and qualitative interview plan are well-detailed, but it is unclear how youth engagement will be incorporated throughout the analysis. Additionally, there is limited information provided about the youth members who will be part of the team.

The target population in Phase 2 will be youths living in Canada ages 10-24 and their family members with an expressed interest in youth engagement in health research. Participants will be recruited via professional networks, social media pages, and email lists. They will also draw on a sample of diverse youth advisors who have participated in previous research with the team. However, there is limited discussion of saturation.

A brief description of the NPA's background is provided. It appears they have significant experience with engagement, and the supporting team has expertise in paediatric healthcare.

The environment, for the research is a bit difficult to discern given limit discussion of the team. Furthermore, although the proposed project is largely related to clinical research activities, it is not clear how community engaged, and culturally rooted approaches will be considered.

Overall, this project has the potential to address important areas of empowered youth, centering youth voices and experiences within the field of health. Phase I was clearly described, however Phase II seemed to be a high level summary lacking key delivery details.

B. Comments on the budget requested and a formal recommendation.

The budget assessment *must not* be factored into the scientific assessment and *must not* influence the rating of applications**.** Please include clear and detailed reasons for any recommended budget cuts.

**Review Type/Type d'évaluation:**	Committee Member 1/Membre de comité 1
**Name of Applicant/Nom du chercheur:**	Munce, Sarah Elizabeth
**Application No./Numéro de demande:**	513340
**Agency/Agence:**	CIHR/IRSC
**Competition/Concours:**	2023-11-08 Catalyst Grant: Healthy Youth/Subvention catalyseur : Jeunes en santé
**Committee/Comité:**	Catalyst Grant : Healthy Youth/Subvention catalyseur : Jeunes en santé
**Title/Titre:**	Youth and Family Specific Engagement in Research (UNITE): A Framework Development Project

Assessment/Évaluation:

No suggested budget changes.

C. Integration of Sex and/or Gender in the Research Proposal

Integrated throughout the proposal.

Sex and gender will be addressed through the scoping review, particularly the focus on sex in relation to health conditions, as indicated in the proposal. In addition, it is indicated that the qualitative interviews will explore sex in relation to health condition and gender in relation to sociocultural gender biases. However, no specific analysis or crosscutting approaches have been identified throughout the proposal.

D. Top (competitive) or bottom (non-competitive) group selection

Middle

E. San Francisco Declaration on Research Assessment

DORA are not clearly addressed.

**Review Type/Type d'évaluation:**	Committee Member 2/Membre de comité 2
**Name of Applicant/Nom du chercheur:**	Munce, Sarah Elizabeth
**Application No./Numéro de demande:**	513340
**Agency/Agence:**	CIHR/IRSC
**Competition/Concours:**	2023-11-08 Catalyst Grant: Healthy Youth/Subvention catalyseur : Jeunes en santé
**Committee/Comité:**	Catalyst Grant : Healthy Youth/Subvention catalyseur : Jeunes en santé
**Title/Titre:**	Youth and Family Specific Engagement in Research (UNITE): A Framework Development Project

Assessment/Évaluation:

Summary:
The objectives of this proposal are: to 1) determine the extent of literature on the application of patient engagement models, theories, frameworks, and guiding principles designed for adults in the context of youth-specific research; and, building on the learnings from the literature, to 2) understand the key components and constructs of these models, theories, frameworks, and guiding principles that are meaningful to youth and their family members (e.g., what does/does not resonate with youth and family members). These objectives will be achieved by conducting a scoping review, followed by qualitative interviews with youth and family members. Collectively, these findings will be foundational to the development of a youth and family specific engagement in research (UNITE) framework, and later, tool to measure meaningful engagement in research for youth and families.

The team is led by an early career investigator, a youth co-Principal Applicant and an early career clinician scientist which is a significant strength. The project is led by a very large team with significant expertise in all areas of relevance to the proposal. The NPA has an excellent Tri-Council funding track record given their career stage.

The topic is timely and of significant relevance to the child and youth health research community and given the partners included has a high likelihood of informing research and potentially practice.

Overall, this is an excellent proposal with a strong team. It is clear and specific and lays out how this research fits within a larger program of research. The review portion of the research is particular strong with much of the protocol already developed. The description of the second qualitative phase of the research is quite short and while it includes all the basic elements that should be included, it is somewhat generic in its description.

Review Type/Type d'évaluation: Committee Member 3/Membre de comité 3
Name of Applicant/Nom du chercheur: Munce, Sarah Elizabeth
Application No./Numéro de demande: 513340
Agency/Agence: CIHR/IRSC
Competition/Concours: 2023-11-08 Catalyst Grant: Healthy Youth/Subvention catalyseur : Jeunes en santé
Committee/Comité: Catalyst Grant : Healthy Youth/Subvention catalyseur : Jeunes en santé
Title/Titre: Youth and Family Specific Engagement in Research (UNITE): A Framework Development Project

---

Assessment/Évaluation:

Summary

The proposed study conducts a scoping review of existing patient engagement frameworks used in the youth health research community and aims to consult diverse youth and caregiver experiences to assess their merits and outline potential areas for improvement. The scoping review and data collection will be conducted by a team of both patient partners and researchers, meaning that patient perspectives will be a part of the process throughout the proposed study.
Overall, it is a very strong application and I would consider it to be highly competitive.

Strengths

The study strongly reflects the values and objectives of this funding opportunity because its proposed analyses and methods directly assess current methods of engaging youth partners and people with lived experience and addressing gaps in already-existing frameworks.
It has the potential to develop and improve methods of collaboration between youth partners, build capacity for involved team members, build capacity within the research community for more meaningful collaborative frameworks in future research, as well as to advance priorities of youth communities in health research.
There are youth members already on the research team, including one of the co-applicants, and includes youth engagement in all stages of the proposed study.
It mentions having multiple youth members on the team and capacity-building measures like peer debriefing to reduce power imbalances and avoid tokenism.
The team has significant and varied experience in both youth engagement, the focus areas, and the proposed methods of study.

Weaknesses

Its potential for cross-sectoral impact with the proposed knowledge dissemination/translation efforts is mainly limited to research community spaces (eg. Inclusion of knowledge mobilization networks /organizations), and could be improved with a social media campaign, workshops, partnerships with relevant youth organizations, etc.
The recruitment process for patient/caregiver participants relies a lot on digital communication, and on previous connection with the research team members, which may introduce bias into the sample (ex. conflict of interest, voluntary response bias for those acquainted via previous research involvement, lack of diverse participants).
The application does not mention how they will manage potential biases in the selection process.

**Review Type/Type d'évaluation:**	Committee Member 3/Membre de comité 3
**Name of Applicant/Nom du chercheur:**	Munce, Sarah Elizabeth
**Application No./Numéro de demande:**	513340
**Agency/Agence:**	CIHR/IRSC
**Competition/Concours:**	2023-11-08 Catalyst Grant: Healthy Youth/Subvention catalyseur : Jeunes en santé
**Committee/Comité:**	Catalyst Grant : Healthy Youth/Subvention catalyseur : Jeunes en santé
**Title/Titre:**	Youth and Family Specific Engagement in Research (UNITE): A Framework Development Project

Assessment/Évaluation:

Additionally, participant interviews are limited to online or phone calls; this improves physical accessibility of the interviews and is a strength, but limiting interviews to only these mediums may make it harder for people of lower socioeconomic status to participate – an alternate option may need to be considered.
The potential issues mentioned above were not discussed or considered in the application, and so it is difficult to assess criteria like the quality of the recruitment strategy, accessibility, strategies for the integration of diverse perspectives in the study.
No specific SGBA approaches are mentioned in the analysis of data, although data on sex and gender will be collected.

Budget -The proposed budget is appropriate for the proposed study.

Review Type/Type d'évaluation: SO Notes /Notes de l'agent scientifique
Name of Applicant/Nom du chercheur: Munce, Sarah Elizabeth
Application No./Numéro de demande: 513340
Agency/Agence: CIHR/IRSC
Competition/Concours: 2023-11-08 Catalyst Grant: Healthy Youth/Subvention catalyseur : Jeunes en santé
Committee/Comité: Catalyst Grant : Healthy Youth/Subvention catalyseur : Jeunes en santé
Title/Titre: Youth and Family Specific Engagement in Research (UNITE): A Framework Development Project

---

Assessment/Évaluation:

PRC: HYI

NPA:Munce, Sarah E

Application Number: 513340

Project Title: Youth and Family Specific Engagement in Research (UNITE): A Framework Development Project

SO Notes begin here:

````````````````````````````````````````````````````````````````````````````````````````````

## Strengths:

This project addresses an important topic and has the potential to inform research in the area of youth engagement in research.

There was youth involvement throughout the project with inclusion of youth voices in the area of patient engagement. Youth will be engaged in both phases of the research. There is strong capacity building for youth researcher members.

Project is feasible and the objectives align with this funding opportunity.

The proposal clearly outlined the two phases. Phase 1 includes a scoping review that is described well. Phase
````````````````````````````````````````````````````````````````````````````````````````````

**Review Type/Type d'évaluation:**	SO Notes /Notes de l'agent scientifique
**Name of Applicant/Nom du chercheur:**	Munce, Sarah Elizabeth
**Application No./Numéro de demande:**	513340
**Agency/Agence:**	CIHR/IRSC
**Competition/Concours:**	2023-11-08 Catalyst Grant: Healthy Youth/Subvention catalyseur : Jeunes en santé
**Committee/Comité:**	Catalyst Grant : Healthy Youth/Subvention catalyseur : Jeunes en santé
**Title/Titre:**	Youth and Family Specific Engagement in Research (UNITE): A Framework Development Project

Assessment/Évaluation:

2 include qualitative interviews with youth.

The literature review for the scoping review will be integrated with the existing information on patient engagement.

This is a strong team with a good funding record.

Sex and gender will be included in the scoping review.

Weaknesses:

While Phase 1 is decribed well, Phase 2 lacks detail. The modality of data collection activities for phase 2 could be barriers to some youth.

There is limited detail on the recruitment strategies and ways to engage divers youth perspectives provided. As the project is focusing on youth aged 10-24 years, innovative approaches beyond interviews to engage youth are missing.

There is no description of ways they might manage bias and potential for power differentials in Phase 2.

The interviews will include sex and gender in the qualitative component however how sex and gender will be accounted for in the analysis is unclear.

**Review Type/Type d'évaluation:**	SO Notes /Notes de l'agent scientifique
**Name of Applicant/Nom du chercheur:**	Munce, Sarah Elizabeth
**Application No./Numéro de demande:**	513340
**Agency/Agence:**	CIHR/IRSC
**Competition/Concours:**	2023-11-08 Catalyst Grant: Healthy Youth/Subvention catalyseur : Jeunes en santé
**Committee/Comité:**	Catalyst Grant : Healthy Youth/Subvention catalyseur : Jeunes en santé
**Title/Titre:**	Youth and Family Specific Engagement in Research (UNITE): A Framework Development Project

Assessment/Évaluation:

The project aims to gather diverse perspectives but clarity of how diversity is considered and will be accounted for is missing.

The KT plan focuses primarily on academic audiences. Engaging youth organizations for KT activities could be useful.

Budget:

****

SO Notes end here.
