# Supplementary figures and images for: Development of a Framework for Youth- and Family-Specific Engagement in Research: Proposal for a Scoping Review and Qualitative Descriptive Study
Source: JMIR Res Protoc. 2025 Mar 28;14:e65733. doi: 10.2196/65733 (PMC11992488; doi:10.2196/65733)

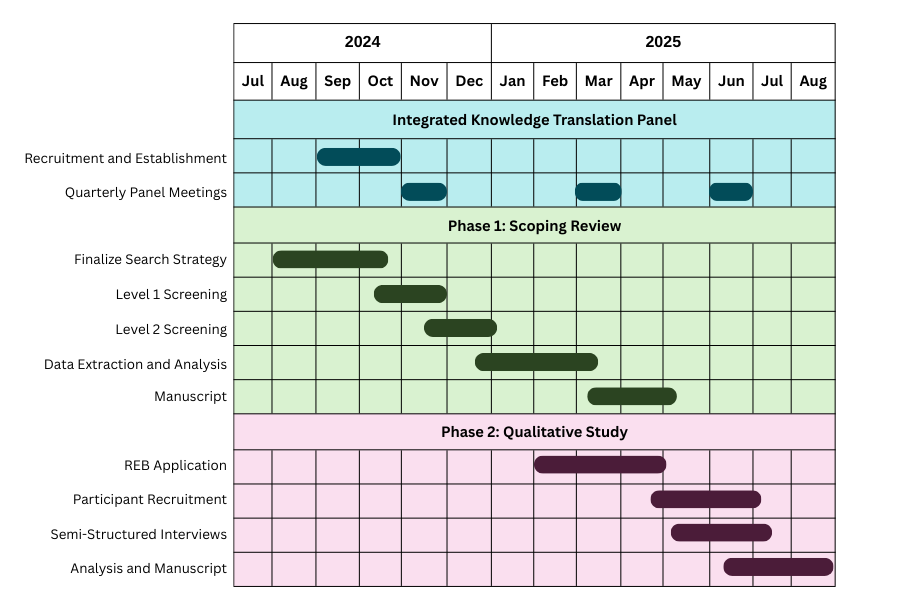

Supplement: Multimedia Appendix 4 [file resprot_v14i1e65733_app4.png]
